# The cross-sectional and longitudinal effect of hyperlipidemia on knee osteoarthritis: Results from the Dongfeng-Tongji cohort in China

**DOI:** 10.1038/s41598-017-10158-8

**Published:** 2017-08-29

**Authors:** Min Zhou, Yanjun Guo, Dongming Wang, Da Shi, Weijin Li, Yuewei Liu, Jing Yuan, Meian He, Xiaomin Zhang, Huan Guo, Tangchun Wu, Weihong Chen

**Affiliations:** 1 Department of Occupational and Environmental Health, School of Public Health, Tongji Medical College, Huazhong University of Science and Technology, Wuhan, 430030 China; 2 Key Laboratory of Environment and Health in Ministry of Education & Ministry of Environmental Protection, and State Key Laboratory of Environmental Health (Incubating), School of Public Health, Tongji Medical College, Huazhong University of Science and Technology, Wuhan, 430030 China; 3 College of Food Science and Technology, Huazhong Agricultural University, Wuhan, 430070 China; 4 Department of Orthopaedic Surgery, Tongji Hospital, Tongji Medical College, Huazhong University of Science and Technology, Wuhan, 430030 China

## Abstract

To quantify the cross-sectional and longitudinal effects of hyperlipidemia on knee osteoarthritis (KOA), we studied 13,906 middle-aged or older participants from the Dongfeng-Tongji cohort. Physical examinations were performed at baseline and follow-up. Knee pain was diagnosed by self-reported pain or stiffness. Clinical KOA was diagnosed from knee pain complains and clinical X-ray radiographs. The prevalence of knee pain and clinical KOA was 39.0% and 6.7% at baseline, respectively. Hyperlipidemia was associated with knee pain (OR 1.34, 1.23–1.45) and clinical KOA (1.34, 1.15–1.55). Compared with the participants without hyperlipidemia or use of lipid-lowering drugs, those with hyperlipidemia but no use of lipid-lowering drugs had higher risks of knee pain (1.28, 1.15–1.43) and clinical KOA (1.20, 0.97–1.48), those with hyperlipidemia and use of lipid-lowering drugs had the highest risks of knee pain (1.40, 1.26–1.56) and clinical KOA (1.45, 1.21–1.75). The risks were not elevated among participants without hyperlipidemia but using lipid-lowering drugs for prevention of other diseases. Furthermore, each 1-unit increase in triglyceride was associated with 9% and 5% increases in the risk of clinical KOA prevalence and clinical KOA onset, respectively. In conclusion, hyperlipidemia is associated with elevated risks of knee pain and clinical KOA among middle-aged or older adults.

## Introduction

Knee osteoarthritis (KOA) is a degenerative arthritis that results from the breakdown of joint cartilage and underlying bone^1^. The results of the Global Burden of Disease Study showed that osteoarthritis cased 12.8 million of YLDs (years lived with disability) and 10.4 million of DALYs (disability-adjusted life years) worldwide in 2013^2, 3^; moreover, KOA is believed to be the leading cause of lower-limb disability in older adults and one of the largest socioeconomic burdensworldwide^4, 5^.

As one of the most common joint diseases worldwide, KOA’s prevalence varies across countries and regions. The global prevalence of KOA was estimated at 3.8% in 2010^6^, and the prevalence ranged from 7.5% to 25.2% in Asia after the year of 2000^7–9^. In China the prevalence of symptomatic KOA was 8.1% in 2012^10^. Such variation in the prevalence of KOA may be due to differences in heredity, anatomy, and case definition.

Although the prevalence of KOA is high, the risk factors remain unclear. Previous research indicates that aging, obesity, and occupational activities are risk factors^11–14^. With aging and obesity, alterations in systemic metabolism become important risk factors for imbalances in bone and cartilage metabolism, believed to be a key factor in the development and progression of KOA. Other factors such as lipid disorders, which may interfere with joint metabolism, have also garnered considerable attention. A few studies have found associations between elevated blood lipids and disturbed differentiation or metabolism of chondrocyte^15–17^. The prevalence of hyperlipidemia have increased dramatically in China^18^, however, the potential effects of hyperlipidemia on KOA have not been adequately evaluated among Chinese. In addition, lipid-lowering drugs which were commonly used by some hyperlipidemias were also reported to have certain impact on the risk of KOA, but the results were inconsistent^19, 20^. Potential effects of lipid-lowering drugs on KOA need further evaluation.

The Dongfeng-Tongji cohort was established in 2008^21^ and was first followed up in 2013. We developed the present study using data concerning knee health and serum lipids at baseline and follow-up. This large sample of middle-aged or older Chinese subjects was used to investigate whether elevated serum lipid levels and the use of lipid-lowering drugs are linked to KOA.

## Results

A total of 13,906 individuals (6,346 males) identified at baseline were included in the cross-sectional analyses. No differences in age, WHR (waist-to-hip ratio), or lifestyle were detected between the included participants and remaining members of the cohort.

The basic characteristics of the included participants were presented in Table 1. The prevalence of knee pain and clinical KOA was 39.0% (N = 5,420) and 6.7% (N = 927), respectively. The age of retirement in China for male and female was 55 and 50 years old, respectively. In the present study, the mean age was 64.7 years, with 365 (4.8%) women younger than 50 years (mean age 47.4 ± 2.0 years) and 146 (2.3%) men younger than 55 years (mean age 49.5 ± 4.1 years), and the reasons likely include serious illness, work injuries and family reason. Participants with knee pain tended to be less physically active than those without such pain. The percentage of current smokers appeared to be lower among the knee pain and clinical KOA cases (12.0% and 8.4%, respectively) than among their unaffected colleagues (17.9% and 16.1%, respectively). The correlation of lipid levels between baseline and follow-up among participants not using lipid-lowering drugs (N = 6,409) were shown in Table 2, and the correlation coefficients were 0.61, 0.63, 0.62, and 0.70 for triglyceride, TC (total cholesterol), LDL-C (low-density lipoprotein cholesterol), and HDL-C (high-density lipoprotein cholesterol), respectively.

**Table 1:** Basic characteristics of the study population at baseline (N = 13, 906).

Variables	Knee pain			Clinical KOA		
	Yes	No	*p-*value	Yes	No	*p-*value
Age (years, mean ± SD)	64.9 ± 8.2	64.6 ± 8.3	0.09	66.3 ± 8.2	64.6 ± 8.2	<0.0001*
Male: <55 (Female: <50)	150(2.8)	361(4.3)	<0.0001*	19(2.1)	492(3.8)	<0.0001*
Male: 55–65 (Female: 50–55)	1377(25.4)	2568(30.3)		182(19.6)	3763(30.0)	
Male: 65–70 (Female: 55–65)	1845(34.0)	2845(33.5)		305(32.9)	4385(33.8)	
Male: ≥70 (Female: ≥65)	2048(37.8)	2712(32.0)		421(45.4)	4339(33.4)	
Male gender (%)	1904(35.1)	4442(52.4)	<0.0001*	281(30.3)	6065(46.7)	<0.0001*
WHR			<0.0001*			<0.0001*
T1: <0.88 (Female: <0.83)	1537(28.6)	2690(32.1)		240(26.0)	3987(31.1)	
T2: 0.88–0.93 (Female: 0.83–0.88)	1864(34.7)	3022(36.0)		309(33.5)	4577(35.6)	
T3: ≥0.93 (Female: ≥0.88)	1969(36.7)	2674(31.9)		373(40.5)	4270(33.3)	
Hyperlipidemia (%)	1493(27.9)	1854(22.1)	<0.0001*	281(31.0)	3066(23.9)	<0.0001*
Hyperlipidemia (%)			<0.0001*			<0.0001*
No, without lipid-lowering drugs	3657(68.4)	6216(74.1)		592(65.3)	9281(72.4)	
Yes, without lipid-lowering drugs	691(12.9)	915(10.9)		116(12.8)	1490(11.6)	
Yes, with lipid-lowering drugs	802(15.0)	939(11.2)		165(18.2)	1576(12.3)	
No, taking lipid-lowering drugs for prevention of other diseases	197(3.7)	314(3.8)		34(3.7)	477(3.7)	
Family history of hyperlipidemia (%)	385(7.1)	470(5.5)	<0.0001*	74(8.0)	781(6.0)	<0.0001*
With comorbidities (%)	3047(56.2)	4360(51.4)	<0.0001*	542(58.5)	6865(52.9)	0.001*
Workload on knee (%)			<0.0001*			0.06
Mild	1074(19.9)	1697(20.1)		190(20.6)	2581(20.0)	
Moderate	2317(42.9)	3407(40.4)		409(44.3)	5315(41.1)	
Severe	2011(37.2)	3338(39.5)		324(35.1)	5025(38.9)	
Exercise (%)	4839(89.3)	7681(90.5)	<0.0001*	828(89.3)	11692(90.1)	0.45
Smoking (%)			<0.0001*			<0.0001*
Non-Smokers	4171(77.1)	5650(66.8)		744(80.4)	9077(70.1)	
Current-Smokers	651(12.0)	1519(17.9)		78(8.4)	2092(16.1)	
Ex-Smokers	590(10.9)	1296(15.3)		104(11.2)	1782(13.8)	
Drinking (%)			<0.0001*			<0.0001*
Non-Drinkers	3857(71.4)	5612(66.3)		668(72.1)	8801(68.0)	
Current-Drinkers	1258(23.3)	2253(26.6)		203(21.9)	3308(25.6)	
Ex-Drinkers	287(5.3)	600(7.1)		55(6.0)	832(6.4)	

SD: standard deviation. WHR: waist-to-hip ratio. Comorbidities included hypertension, diabetes, coronary heart disease, myocardial infarction, stroke, or tumors. *p-value < 0.05.

**Table 2:** The correlation of lipid levels between baseline and follow-up among participants without use of lipid-lowering drugs (N = 6,409).

Variables	Baseline lipid levels Median (5%–95% quantile)	Follow-up lipid levels Median (5%–95% quantile)	Spearman correlation coefficient (95% CI)	*p*-value
TG	1.11 (0.50–2.74)	1.23 (0.69–2.97)	0.61 (0.60–0.63)	<0.0001*
TC	5.10 (3.75–6.71)	4.87 (3.42–6.57)	0.63 (0.62–0.65)	<0.0001*
LDL-C	2.93 (1.80–4.25)	2.75 (1.50–4.14)	0.62 (0.60–0.63)	<0.0001*
HDL-C	1.43 (0.97–2.13)	1.38 (0.94–2.05)	0.70 (0.68–0.71)	<0.0001*

TC: total cholesterol. TG: triglyceride. LDL-C: low-density lipoprotein cholesterol. HDL-C: high-density lipoprotein cholesterol. *p-value < 0.05.

Risk factors for knee pain and clinical KOA identified in this study were advanced age, elevated WHR, physical workload, and hyperlipidemia (Table 3). The adjusted ORs (odds ratios) of knee pain and clinical KOA among hyperlipidemia cases were 1.34(1.23–1.45) and 1.34(1.15–1.55), respectively. Additionally, a family history of hyperlipidemia was a risk factor for knee pain (1.26, 1.09–1.45) and clinical KOA (1.45, 1.12–1.87).

**Table 3:** Odds ratios (OR) of risk factors for knee pain and clinical KOA.

Variables	Knee pain			Clinical KOA		
	Crude OR	Adjusted-OR^1^	Adjusted-OR^2^	Crude OR	Adjusted-OR^1^	Adjusted-OR^2^
Age (years)						
Male: 55–65 (Female: 50–55)	1	1		1	1	
Male: <55 (Female: <50)	0.78(0.63–0.95)*	0.66(0.54–0.81)*		0.80(0.49–1.29)	0.70(0.43–1.13)	
Male: 65–70 (Female: 55–65)	1.21(1.11–1.32)*	1.17(1.07–1.28)*		1.44(1.19–1.74)*	1.39(1.15–1.68)*	
Male: ≥70 (Female: ≥65)	1.41(1.29–1.54)*	1.36(1.25–1.49)*		2.01(1.68–2.40)*	1.94(1.62–2.32)*	
Female gender	2.03(1.89–2.18)*	—		2.02(1.75–2.33)*	—	
WHR						
T1: <0.88 (Female: <0.83)	1	1		1	1	
T2: 0.88–0.93 (Female: 0.83–0.88)	1.08(0.99–1.18)	1.11(1.02–1.21)*		1.12(0.94–1.33)	1.15(0.97–1.37)	
T3: ≥0.93 (Female: ≥0.88)	1.29(1.18–1.40)*	1.28(1.18–1.40)*		1.45(1.23–1.72)*	1.44(1.22–1.70)*	
Physical workload						
Mild	1	1		1	1	
Moderate	1.08(0.98–1.18)	1.03(0.94–1.13)		1.04(0.88–1.25)	1.01(0.84–1.20)	
Severe	0.95(0.87–1.05)	1.17(1.06–1.29)*		0.88(0.73–1.05)	1.06(0.88–1.28)	
Hyperlipidemia						
No	1	1	1	1	1	1
Yes	1.36(1.26–1.48)*	1.38(1.28–1.50)*	1.34(1.23–1.45)*	1.43(1.23–1.65)*	1.44(1.24–1.67)*	1.34(1.15–1.55)*
Hyperlipidemia						
No, without lipid-lowering drugs	1	1	1	1	1	1
Yes, without lipid-lowering drugs	1.28(1.15–1.43)*	1.30(1.16–1.44)*	1.28(1.15–1.43)*	1.22(0.99–1.50)	1.23(1.01–1.52)*	1.20(0.97–1.48)
Yes, with lipid-lowering drugs	1.45(1.31–1.61)*	1.47(1.33–1.63)*	1.40(1.26–1.56)*	1.64(1.37–1.97)*	1.67(1.39–2.00)*	1.45(1.21–1.75)*
No, taking lipid-lowering drugs for prevention of other diseases	1.07(0.89–1.28)	1.07(0.89–1.28)	1.04(0.80–1.26)	1.12(0.78–1.60)	1.14(0.80–1.63)	0.99(0.69–1.43)
Family history of hyperlipidemia						
No	1	1	1	1	1	1
Yes	1.30(1.14–1.50)*	1.28(1.11–1.47)*	1.26(1.09–1.45)*	1.36(1.06–1.74)*	1.34(1.05–1.73)*	1.45(1.12–1.87)*

WHR: waist-to-hip ratio. *p-value < 0.05. Adjusted-OR^1^: adjusted for gender. Adjusted-OR^2^: adjusted for age (as a continuous variable), WHR (as a continuous variable), gender, physical workload, physical exercise, smoking, and drinking.

The results of stratified analyses by lipid-lowering drugs were shown in Table 3. Compared with participants without hyperlipidemia or use of lipid-lowering drugs, those with hyperlipidemia but no use of lipid-lowering drugs had higher risks of knee pain (1.28, 1.15–1.43) and clinical KOA (1.20, 0.97–1.48), and those with hyperlipidemia and use of lipid-lowering drug had the highest risks of knee pain (1.40, 1.26–1.56) and clinical KOA (1.45, 1.21–1.75). Notably, the ORs of knee pain (1.04, 0.80–1.26) and clinical KOA (0.99, 0.69–1.43) were not significantly elevated among the participants without hyperlipidemia but using lipid-lowering drugs for prevention of other diseases.

In stratified analyses by WHR, risk of knee pain was significantly elevated among hyperlipidemia cases with or without use of lipid-lowering drugs in all WHR-groups (Table 4). The significantly elevated risk of clinical KOA was observed mainly in the high-WHR groups.

**Table 4:** Associations between hyperlipidemia and knee pain and clinical KOA stratified by WHR.

Variables	Knee pain			Clinical KOA		
	T1 (N = 4227)	T2 (N = 4886)	T3 (N = 4643)	T1 (N = 4227)	T2 (N = 4886)	T3 (N = 4643)
Hyperlipidemia						
No	1	1	1	1	1	1
Yes	1.38(1.17–1.63)*	1.31(1.14–1.50)*	1.35(1.18–1.54)*	1.24(0.90–1.72)	1.28(0.99–1.66)	1.41(1.12–1.77)*
Hyperlipidemia						
No, without lipid-lowering drugs	1	1	1	1	1	1
Yes, without lipid-lowering drugs	1.21(0.97–1.51)	1.29(1.07–1.55)*	1.31(1.10–1.57)*	1.08(0.69–1.71)	1.03(0.70–1.50)	1.38(1.02–1.88)*
Yes, with lipid-lowering drugs	1.63(1.30–2.05)*	1.36(1.14–1.63)*	1.35(1.14–1.59)*	1.41(0.93–2.14)	1.44(1.06–1.97)*	1.48(1.12–1.96)*
No, with lipid-lowering drugs for prevention of other diseases	1.16(0.80–1.67)	1.24(0.90–1.72)	0.83(0.62–1.13)	1.05(0.50–2.21)	0.62(0.28–1.34)	1.28(0.78–2.11)
Family history of hyperlipidemia						
No	1	1	1	1	1	1
Yes	1.11(0.85–1.43)	1.12(0.88–1.43)	1.63(1.27–2.09)*	1.14(0.68–1.92)	1.30(0.82–2.05)	1.88(1.27–2.76)*

WHR: waist-to-hip ratio. T1: WHR <0.88 (female:  <0.83); T2: WHR 0.88–0.93 (female: 0.83–0.88); T3: WHR ≥0.93 (female: ≥0.88). Adjusted for age (as a continuous variable), gender, physical workload, physical exercise, smoking, and drinking. *p-value < 0.05.

The associations between specific blood lipids and either knee pain or clinical KOA were shown in Table 5. Compared with subjects with normal lipids and no use of lipid-lowering drugs, ORs of knee pain (1.36–1.43) and clinical KOA (1.49–1.57) were significantly higher in all groups containing subjects with hyperlipidemia and use of lipid-lowering drugs. Each 1-unit increase in triglyceride was associated with 5% and 9% increases in the risks of knee pain and clinical KOA, respectively. Additionally, each 1-unit increase in TC and LDL-C was associated with 8% and 6% increases in the risk of knee pain, respectively.

**Table 5:** Odds ratios (OR) of knee pain and clinical KOA associated with serum lipid levels in the cross-sectional analysis (N = 13,906).

Variables	ORs increase per 1-unit increase in serum lipid levels	ORs for the levels of serum lipid versus the normal group			
		Group 1	Group 2	Group 3: With hyperlipidemia and use of lipid-lowering drugs	Group 4: Without hyperlipidemia but taking lipid-lowering drugs for prevention of other diseases
Knee pain					
TC	1.08 (1.05–1.12)*	Reference	1.07 (0.99–1.16)	1.42 (1.28–1.58)*	1.04 (0.86–1.25)
TG	1.05 (1.01–1.08)*	Reference	1.11 (1.02–1.21)*	1.43 (1.28–1.58)*	1.04 (0.87–1.26)
LDL-C	1.06 (1.02–1.11)*	Reference	1.07 (0.98–1.18)	1.40 (1.27–1.56)*	1.03 (0.86–1.24)
HDL-C	1.03 (0.93–1.14)	Reference	0.90 (0.80–1.01)	1.36 (1.23–1.51)*	1.00 (0.83–1.20)
Clinical KOA					
TC	1.06 (0.99–1.13)	Reference	1.03 (0.88–1.20)	1.54 (1.28–1.85)*	1.01 (0.70–1.46)
TG	1.09 (1.04–1.14)*	Reference	1.12 (0.94–1.32)	1.57 (1.30–1.88)*	1.04 (0.72–1.49)
LDL-C	0.98 (0.91–1.07)	Reference	1.03 (0.86–1.24)	1.53 (1.28–1.83)*	1.01 (0.70–1.45)
HDL-C	0.97 (0.80–1.19)	Reference	0.85 (0.66–1.09)	1.49 (1.24–1.78)*	0.98 (0.69–1.41)

TC: total cholesterol. TG: triglyceride. LDL-C: low-density lipoprotein cholesterol. HDL-C: high-density lipoprotein cholesterol.

TC: group 1, <5.18 mmol/L and without lipid-lowering drugs; group 2, ≥5.18 mmol/L and without lipid-lowering drugs.

TG: group 1, <1.70 mmol/L and without lipid-lowering drugs; group 2, ≥1.70 mmol/L and without lipid-lowering drugs.

LDL-C: group 1, <3.37 mmol/L and without lipid-lowering drugs; group 2, ≥3.37 mmol/L and without lipid-lowering drugs.

HDL-C: group 1, ≥1.04 mmol/L and without lipid-lowering drugs; group 2, <1.04 mmol/L and without lipid-lowering drugs.

Adjusted for age (as a continuous variable), WHR (as a continuous variable), and gender. * *p*-value < 0.05.

The association between hyperlipidemia and clinical KOA was further investigated longitudinally. During 4.91 (standard deviation 0.49) years of follow-up, 361 clinical KOA patients were newly diagnosed. Compared with participants without hyperlipidemia or use of lipid-lowering drugs, the covariates-adjusted HR (hazard ratio) of clinical KOA was significant for all hyperlipidemia cases. Risk of clinical KOA was highest among participants with hyperlipidemia and use of lipid-lowering drugs (HR 1.40, 1.02–1.90). As shown in Table 6, each 1-unit increase in triglyceride was associated with a 5% increase in the risk of developing clinical KOA. Compared to individuals with normal triglyceride and no use of lipid-lowering drugs, the HR for clinical KOA was 1.58(1.20–2.09) among those with hyperlipidemia and taking lipid-lowering drugs. The HR for clinical KOA among those without hyperlipidemia but taking lipid-lowering drugs for prevention of other diseases was elevated without significance (HR 1.34, 0.63–2.86).

**Table 6:** Hazard ratios (HRs) of clinical KOA associated with serum lipids levels in the cohort analysis (N = 9062), 2008–2013.

Variables	HRs increase per 1-unit increase in serum lipids levels	HRs for the levels of serum lipids versus the normal group			
		Group 1	Group 2	Group 3: With hyperlipidemia and use of lipid-lowering drugs	Group 4: Without hyperlipidemia but taking lipid-lowering drugs for prevention of other diseases
TC	1.04(0.94–1.16)	Reference	0.95(0.75–1.20)	1.53(1.14–2.04)*	1.30(0.61–2.77)
TG	1.05(1.01–1.10)*	Reference	1.05(0.79–1.38)	1.58(1.20–2.09)*	1.34(0.63–2.86)
LDL-C	1.00(0.90–1.17)	Reference	1.10(0.86–1.41)	1.61(1.21–2.14)*	1.37(0.64–2.92)
HDL-C	1.01(0.75–1.36)	Reference	0.93(0.65–1.34)	1.55(1.18–2.04)*	1.32(0.62–2.80)

TC: total cholesterol. TG: triglyceride. LDL-C: low density lipoprotein cholesterol. HDL-C: high density lipoprotein cholesterol.

TC: group 1, <5.18 mmol/L and without lipid-lowering drugs; group 2, ≥5.18 mmol/L and without lipid-lowering drugs.

TG: group 1, <1.70 mmol/L and without lipid-lowering drugs; group 2, ≥1.70 mmol/L and without lipid-lowering drugs.

LDL-C: group 1, <3.37 mmol/L and without lipid-lowering drugs; group 2, ≥3.37 mmol/L and without lipid-lowering drugs.

HDL-C: group 1, ≥1.04 mmol/L and without lipid-lowering drugs; group 2, <1.04 mmol/L and without lipid-lowering drugs.

Adjusted for age (as a continuous variable), WHR (as a continuous variable), and gender. *p-value < 0.05.

## Discussion

In the present study, we identified a positive association between hyperlipidemia and elevated risks of knee pain and clinical KOA in middle-aged or older adults. Based on cross-sectional and longitudinal analyses, a positive dose-response relationship was identified between triglyceride and clinical KOA. Moreover, our results indicate that the influence of lipid-lowering drugs on knee pain and clinical KOA is limited.

These findings have notable implications for public health, because the relatively high prevalence of KOA seriously affects quality of life for older adults, and hyperlipidemia may be prevented through diet and physical exercise. Only a few studies have investigated the relationship between hyperlipidemia and osteoarthritis. Dahaghin and colleagues reported no significant association between serum lipids and OA of the hand^22^. However, the Ulm Osteoarthritis Study involving 809 patients with knee or hip joint replacements due to OA indicated that hypercholesterolemia and high serum cholesterol were risk factors for OA^23^. Their results conflicted and the assessment of the effects of hyperlipidemia on KOA in larger populations is necessary. In our cross-sectional analyses of 13,906 participants, risks of knee pain and clinical KOA were significantly higher among hyperlipidemia cases. Similar results were obtained when the analyses were stratified by WHR. In the longitudinal analysis, risk of clinical KOA was higher among participants with hyperlipidemia. Further results were that serum triglyceride linked to KOA risk more closely than serum cholesterols did. Each 1-unit increase in serum triglyceride was associated with a 9% increase in the risk of clinical KOA prevalence and a 5% increase in the risk of clinical KOA onset.

Lipid-lowering drugs have been reported to play a role in the development of KOA, though that role remains unclear. Clockaerts and colleagues demonstrated that lipid-lowering drugs were associated with slower KOA progression^20^. However, Riddle and colleagues failed to demonstrate any positive association between lipid-lowering drugs and decreased KOA risk^19^. In the present study, we did not observe an increased risk of knee pain or clinical KOA among the group of participants without hyperlipidemia but taking lipid-lowering drugs for prevention of other diseases, although the number of subjects in this group (N = 511) was relatively small. Conversely, among participants with hyperlipidemia, risks for knee pain and clinical KOA were higher among those taking lipid-lowering drugs than among those not taking such drugs. Possible explanations for this finding may be that the participants with hyperlipidemia and use of lipid-lowering drugs had higher serum lipids before pharmacotherapy and longer course of hyperlipidemia. Our results suggest that hyperlipidemia may be an independent risk factor for KOA, and that the effect of lipid-lowering drugs on this association is limited.

To our knowledge, this is the first study to evaluate hyperlipidemia as a risk factor for KOA in a large population. Though the reasons underlying this increased risk are not sufficiently clear, several factors may contribute to this association, the first of which may be central obesity. In the WHR-stratified analyses, hyperlipidemia was positively associated with elevated risks of knee pain and clinical KOA in the higher-WHR groups, while the association was weaker in the lower-WHR group. Moreover, elevated serum triglyceride, which is one index of the metabolic disorders following central obesity^24^, was strongly associated with elevated KOA risk. Second, hyperlipidemia is a sign of disorders of lipid metabolism, which could disturb the balance between lipid and bone or cartilage metabolism. Adipocytes share a mesenchymal stem cell precursor with osteoblasts and chondrocytes (i.e. musculoskeletal cells)^25^. Elevated lipids level up-regulate proliferation of adipocytes, which could competitively inhabit proliferation and differentiation of musculoskeletal cells. This phenomenon has been observed by published experiments. Diascro and colleagues found that fatty acids can initiate the switch from osteoblasts to adipocyte-like cells *in vitro* ^26^. Aspden and colleagues reported that elevated lipids level might implicate the formation of musculoskeletal cells from their stromal precursors^27^. Third, inflammatory factors might partially explain the association. With hyperlipidemia, fatty acids and cholesterol crystals increase, possibly activating the Nalp3-inflammasome and leading to increased release of IL-1β, one of the critical pro-inflammatory cytokines involved in the development and progression of KOA^28–30^. Fourth, subchondral ischemia might be another reason involved. Lipids deposit in cartilage and reduce blood flow, and then leads to ischemia and ultimately bone dystrophy^31, 32^.

The strengths of our study include the large sample size, detailed information regarding demographics, lifestyle, medical history, and occupational history for each participant, and examinations of the knee joint and measurement of blood lipids. However, this study also has several limitations. First, we did not provide knee X-ray examination for the participants although all participants could take knee X-ray examination at any time if they have knee discomfort and the company will pay for such examination. And we could not rule out the obese participant or those with hyperlipidemia would more likely to take medical consultation. Second, we were unable to collect information regarding the types and doses of lipid-lowering drugs. Nevertheless, previous studies have reported that commonly used lipid-lowering drugs showed similar effects on KOA^33–35^. Third, we did not collect information regarding dietary patterns and were unable to evaluate the possible confounding influence of this factor. However, considering that all participants were retired from the same large company in the same city, their dietary patterns were likely to be relatively homogenous. Forth, although information regarding physical activity was collected over the past five years, the individuals may have changed their physical activity levels over time. Fifth, we did not evaluate the effect of osteoporosis or serum calcium level on KOA.

In conclusion, our results indicate that hyperlipidemia may be an independent risk factor for knee pain and clinical KOA among middle-aged or older adults. These findings have substantial implications for the prevention of KOA through the reduction of blood lipid levels.

## Methods

### Study Population

The participants were from the Dongfeng-Tongji cohort, which has been described previously^21^. Briefly, the retired employees of the Dongfeng Motor Corporation who agreed to answer the questionnaires and provided baseline blood samples starting in 2008 were included. The first follow-up was conducted in 2013. Questionnaire survey and physical examination were conducted at baseline and during the follow-up. Standard questionnaires were administered by trained interviewers through face-to-face interviews to collect information on demographics, medical history, occupational history, and lifestyle. After the interviews, all participants underwent physical examinations. A group of 14,438 participants, i.e. all of the participants that underwent physical examinations at the Central Hospital of the Dongfeng Motor Corporation, were additionally questioned about their knee health status. After excluding individuals with missing data regarding knee health status (N = 182), with knee surgery caused by accidental injury which was associated with secondary knee osteoarthritis (N = 247), or with history of rheumatoid arthritis (N = 103), data from 13,906 participants was analyzed in a cross-sectional study. Of those participants, 9,335 with baseline information and were followed up. After the exclusion of participants with clinical KOA at baseline (N = 273), 9,062 participants were included in the longitudinal analyses (Supplemental Fig. S1). For the longitudinal analysis, the responders had older age (66.9 ± 7.3 years) than the non-responders (60.7 ± 8.4 years), but other socio-demographic characteristics were similar between the responders and non-responders.

### Ethics Statement

This study was approved by the Medical Ethics Committee of Dongfeng General Hospital, Dongfeng Motor Corporation, and the School of Public Health, Tongji Medical College, Huazhong University of Science and Technology (the review board study approval number: 2010–16). The methods were carried out in accordance with the relevant guidelines and regulations. All participants provided written informed consent.

### Assessment of Knee Pain and Clinical KOA

The information regarding knee pain was collected by trained investigators. Knee pain was diagnosed when an individual’s unilateral or bilateral knee met at least one of the three following criteria: (1) pain within the past 12 months; (2) persistent pain within the past week; or (3) stiffness within the past 12 months.

Information regarding self-reported clinical diagnosed knee osteoarthritis and the year in which the diagnosed was first made was collected from the questionnaires and confirmed by insurance records and treatment information. Clinical KOA was diagnosed only if the patient both had at least one of the three knee pain complains and the bilateral weight-bearing anteroposterior X-ray radiographs showed a Kellgren & Lawrence grade ≥2 in at least one knee^36^.

### Assessment of Hyperlipidemia

Fasting serum lipids, i.e., total cholesterol (TC), triglyceride, high-density lipoprotein cholesterol (HDL-C), and low-density lipoprotein cholesterol (LDL-C) measured to the nearest 0.01 mmol/L (ARCHITECT CI8200, Abbott, USA), were measured for each participant at baseline and during follow-up. Information regarding hyperlipidemia history and the use of lipid-lowering drugs was collected from questionnaires at baseline and follow-up.

We defined hyperlipidemia according to Chinese guidelines on the prevention and treatment of dyslipidemia in adults^37^, which are similar to the criteria adopted by WHO. Hyperlipidemia was diagnosed in participants who fulfilled at least one of the following criteria: (1) TC ≥ 5.18 mmol/L or triglyceride ≥1.70 mmol/L; or (2) self-reported clinically diagnosed hyperlipidemia.

To understand the potential impact of lipid-lowering drugs on KOA, all participants were further classified into four subgroups: no hyperlipidemia or use of lipid-lowering drugs; hyperlipidemia but no use of lipid-lowering drugs; hyperlipidemia and use of lipid-lowering drugs; and without hyperlipidemia but taking lipid-lowering drugs for prevention of other diseases (such as cardiovascular disease and diabetes).

### Assessment of Covariates

Waist and hip circumference were measured to the nearest 0.1 cm with participants wearing light indoor clothing. The waist-to-hip ratio (WHR) was calculated and categorized into three subgroups according to the tertile points: <0.88, 0.88–0.93, ≥0.93 for men and <0.83, 0.88–0.93, ≥0.93 for women.

Information regarding smoking status (current, former, never), drinking status (current, former, never), and physical exercise was collected from the questionnaires. Participants who had been smoking as much as one cigarette per day for at least 6 months were considered current smokers, and those who had been drinking alcohol as often as once per week for at least 6 months were considered current drinkers. Physical exercise was defined as regular exercise of at least 20 minutes per day over the past 6 months.

We also collected data concerning occupational history from the questionnaires. The physical workload on the knees was evaluated according to the longest job that the participant had taken, and it was divided into three subgroups (severe, moderate, and mild) according to the descriptions of the work content and work postures^38–40^.

### Statistical Analysis

Numerical variables were presented as mean and standard deviation and were compared using Student’s t-test, whereas categorical variables were reported as percentages and were compared using Chi-square test. Correlation analysis was conducted between the lipid level at baseline and that during follow-up among participants without use of lipid-lowering drugs. Univariable logistic regression analyses were used to calculate the crude odds ratios (ORs) and 95% confidence intervals (CIs) for KOA and explanatory variables. The adjusted ORs of hyperlipidemia and KOA were calculated using multivariable logistic regression analyses (including covariates of age, WHR, gender, physical workload, physical exercise, smoking, and drinking). Stratified analyses by WHR were also conducted to estimate the associations between hyperlipidemia and KOA at different WHR levels. In the longitudinal analyses, the hazard ratios (HRs) and 95% CIs for clinical KOA and hyperlipidemia were calculated using Cox regression models, in which the time variable for incident clinical KOA was the period from when the participant entered the cohort at baseline to when the participant was followed up to diagnosis with clinical KOA or 2013. A two-sided *p*-value < 0.05 was regarded as statistically significant. All statistical analyses were performed using SAS 9.4 software (SAS Institute, Cary, NC).

## Electronic supplementary material

Supplementary Information
