# Supplementary material for: The cross-sectional and longitudinal effect of hyperlipidemia on knee osteoarthritis: Results from the Dongfeng-Tongji cohort in China
Source: Sci Rep. 2017 Aug 29;7:9739. doi: 10.1038/s41598-017-10158-8 (PMC5575029; doi:10.1038/s41598-017-10158-8)
Supplement: Supplementary file 1 — Supplementary Information [file 41598_2017_10158_MOESM1_ESM.pdf]

The cross-sectional and longitudinal effect of hyperlipidemia on knee osteoarthritis: Results from the Dongfeng-Tongji cohort in China

Min Zhou, Yanjun Guo, Dongming Wang, Da Shi, Weijin Li, Yuewei Liu, Jing Yuan, Meian He, Xiaomin Zhang, Huan Guo, Tangchun Wu, and Weihong Chen

Supplemental Table S1. Hazard ratios (HRs) of clinical KOA associated with changes of serum lipids levels between baseline and follow-up among participants with no use of lipid-lowering drugs (N=6,409).

Variables	Changes of serum lipids levels (as a numerical variable)	Changes of serum lipids levels (as a categorical variable)		
		Lower tertile	Middle tertile	Upper tertile
TG	1.12 (1.04-1.22)*	Reference	0.94 (0.67-1.33)	1.33 (0.96-1.83)
TC	1.13 (0.95-1.34)	Reference	1.24 (0.88-1.75)	1.38 (0.98-1.93)
LDL-C	0.94 (0.77-1.15)	Reference	1.24 (0.89-1.73)	1.17 (0.83-1.64)
HDL-C	1.10 (0.67-1.80)	Reference	0.94 (0.68-1.32)	1.08 (0.78-1.48)

TG: triglyceride. TC: total cholesterol. LDL-C: low-density lipoprotein cholesterol. HDL-C: high-density lipoprotein cholesterol.
TG: lower tertile <-0.06, middle tertile -0.06 to 0.31, upper tertile ≥0.31.
TC: lower tertile <-0.53, middle tertile -0.53 to 0.09, upper tertile ≥0.09.
LDL-C: lower tertile <-0.41, middle tertile -0.41 to 0.08, upper tertile ≥0.08.
HDL-C: lower tertile <-0.15, middle tertile -0.15 to 0.04, upper tertile ≥0.04.
Adjusted for age (as a numerical variable), WHR (as a numerical variable), and gender. * *p*-value <0.05.

Supplementary Table S2. Odds ratios (ORs) of risk factors for knee pain and clinical KOA.

Variables	Without comorbidities		With comorbidities	
	Knee pain	Clinical KOA	Knee pain	Clinical KOA
Hyperlipidemia				
No	1	1	1	1
Yes	1.48(1.26-1.75)*	1.68(1.26-2.23)*	1.22(1.11-1.35)*	1.22(1.01-1.46)*
Hyperlipidemia				
No, without lipid-lowering drugs	1	1	1	1
Yes, without lipid-lowering drugs	1.46(1.21-1.76)*	1.53(1.09-2.14)*	1.14(0.99-1.31)	1.03(0.79-1.35)
Yes, with lipid-lowering drugs	1.58(1.15-2.16)*	2.17(1.34-3.51)*	1.27(1.13-1.44)*	1.31(1.06-1.62)*
No, taking lipid-lowering drugs for prevention of other disease	-	-	0.93(0.76-1.15)	0.86(0.57-1.30)
Family history of hyperlipidemia				
No	1	1	1	1
Yes	1.42(1.18-1.72)*	1.87(1.35-2.59)*	1.05(0.89-1.24)	1.19(0.89-1.59)

Adjusted for age (as a continuous variable), WHR (as a continuous variable), gender, physical workload, physical exercise, smoking, and drinking. Comorbidities included hypertension, diabetes, coronary heart disease, myocardial infarction, stroke, or tumors. * p-value <0.05.

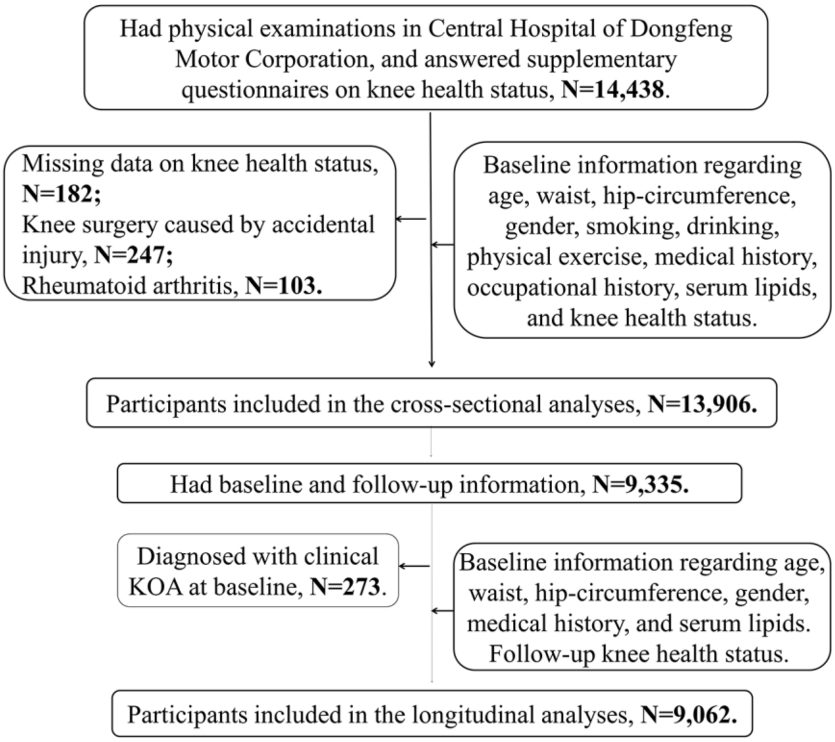

Supplemental Figure S1. Participant flowchart.
